# Evidence of Help-Seeking Behaviors Among Black Women Under Community Supervision in New York City: A Plea for Culturally Tailored Intimate Partner Violence Interventions

**DOI:** 10.1089/whr.2022.0004

**Published:** 2022-11-01

**Authors:** Dawn Goddard-Eckrich, Brandy F. Henry, Srishti Sardana, Brittany V. Thomas, Ariel Richer, Timothy Hunt, Mingway Chang, Karen Johnson, Louisa Gilbert

**Affiliations:** ^1^ The Social Intervention Group, Columbia University, School of Social Work, New York, New York, USA.; ^2^ Rehabilitation and Human Services, Educational Psychology, Counseling, and Special Education, College of Education, Consortium on Substance Use and Addiction, Social Science Research Institute, The Pennsylvania State University, University Park, Pennsylvania, USA.; ^3^ Clinical Psychology, Teachers College, Columbia University, New York, New York, USA.; ^4^ University of Alabama, School of Social Work, Little Hall Room 2005, Tuscaloosa, Alabama, USA.

**Keywords:** intimate partner violence, Black/African American women, social support, community supervision

## Abstract

**Background:**

Black women involved in the legal system disproportionately experience intimate partner violence (IPV); however, current research does not satisfactorily describe the risk and protective factors associated with IPV among Black women under community supervision.

**Methods:**

We conducted a subgroup analysis of Black women (*N* = 128) using data from a randomized controlled trial that evaluated the feasibility and efficacy of two IPV screening and prevention programs for women under community supervision. Participants in the original study were randomized into two IPV prevention conditions—computerized or case manager Women Initiating New Goals of Safety (WINGS). In this study, we examine the effects of that study's two conditions on linkage to IPV services and secondary outcomes, specifically among Black participants who experienced physical, sexual, and psychological IPV.

**Results:**

Both conditions showed significant reductions in days of substance use abstinence over the 3-month period among Black women who experienced sexual or verbal IPV. Participants in the case manager arm were 14 times more likely to receive IPV services in the past 90 days—from baseline to the 3-month follow-up (adjusted odds ratio = 14.45, 95% confidence interval [CI] = 1.25 to 166.51, *p* = 0.032). Participants in the computerized arm were significantly more likely to report receiving social support from baseline to the 3-month follow-up assessment (regression coefficient [*b*] = 2.27, 95% CI = 0.43 to 4.11, *p* = 0.015).

**Conclusions:**

Although both conditions showed significant reductions in the number of days of abstinence from substance use among this subgroup of Black women, the findings showed differential effectiveness between the computerized WINGS arm and the case manager WINGS arm in improving social support and linkage to services. These findings may indicate that different modalities of WINGS may work better for specific activities and point to the need for a hybrid format that optimizes the use of distinct modalities for delivering activities.

## Introduction

Intimate partner violence (IPV) is a significant public health concern in the United States, affecting one in four women each year.^1,2^ Recently, the prevalence of IPV has increased due to the compounded health, social, and economic distress of the COVID-19 pandemic.^3^ When Black women experience IPV—and 40% of them have experienced some form of sexual or physical IPV—the incidents occur more frequently and with greater severity compared with non-Hispanic White women.^4–7^ Additionally, women with a criminal legal system history face significantly more instances of and more severe IPV victimization when compared with women without experience in the criminal legal system.^8–11^

In addition to an increased prevalence, frequency, and severity of experiencing IPV, Black women experience race-associated health disparities in accessing IPV services, which are associated with an increased risk of developing mental illness and reporting fair or poor health.^8,10,12–15^ Additionally, IPV has been linked to structural disadvantages and social determinants of health, including access to health care, poor health outcomes, and substance use disorders (SUDs).^16^

Although IPV research is increasingly attentive to intersectionality,^17^ we know little about the specific protective factors that may reduce IPV among Black women under community supervision who have experienced IPV and have a history of substance use.

### IPV and social support

While Black women face disproportionate IPV prevalence, severity, and frequency, they also have robust adaptive coping strategies, including some forms of social support,^18,19^ which may positively affect their well-being, enable them to protect themselves from IPV, and reduce substance use.^20,21^ Specifically, social networking and support from others compose the social support construct^22,23^ and are prime examples of an adaptive coping strategy in response to stressors associated with IPV.^24^ Furthermore, those with more social support have reported higher self-esteem and deploy more positive coping skills.^25^

Therefore, it is crucial to identify intervention strategies and modalities that may enhance social support, which may then be applied to help Black women to protect themselves from IPV. In contrast, not having a social support network can lead to social isolation, which can harm psychological and physical health and manifest in depression, adverse health behaviors, and mortality.^26^

### Study aims

Black Americans comprise ∼39% of the U.S. population involved in the criminal legal system, including community supervision programs, although they make up <13% of the U.S. population.^27,28^ Despite overrepresentation in the criminal legal system and IPV prevalence rates, only a few studies have examined ways to prevent IPV and improve IPV outcomes among Black women under community supervision with a history of substance use and IPV.

To address these gaps in knowledge, this study aims to evaluate the effectiveness of different modalities (computerized self-paced vs. case manager) of a brief, evidence-based IPV prevention intervention entitled Women Initiating New Goals of Safety (WINGS) on (1) increasing social support; (2) increasing linkage to IPV services; (3) increasing IPV self-efficacy; and (4) increasing the numbers of days of abstinence from drug use. The study focuses on Black women in community supervision programs who have experienced IPV and have a substance use history.

We analyzed data from a parent study titled Women Initiating New Goals of Safety (WINGS) for a subgroup of Black participants who experienced physical, sexual, and psychological IPV. Examining only Black women allows us to identify study effects on this population.

## Materials and Methods

### Overview of the parent study design

WINGS was a randomized controlled trial funded by the National Institute on Drug Abuse (NIDA). The study focused on women who use drugs or engage in binge drinking and were under community supervision in New York City. The study compared two interventions related to IPV screening and prevention: (1) a single-session, self-paced, computerized IPV screening plus a brief IPV prevention tool (computerized WINGS) and (2) the same screening plus IPV prevention material conducted by study case managers (case manager WINGS).

The four primary outcomes measured were social support, IPV service linkage, IPV self-efficacy, and substance use. Across both study arms (computerized and case manager), the physical, sexual, or psychological IPV rates in the year before the screening activity were about 77% (77.3% for computerized and 77.7% for case manager). At the 3-month follow-up, both the computerized and case manager WINGS conditions saw improvements in all four study outcomes: increases in social support, IPV self-efficacy, linkage to care for IPV services, and abstinence from drug use.^33^

Project WINGS was conducted in collaboration with the New York City Department of Probation, the Center for Court Innovation, and Bronx Community Solutions. Additionally, a Community Collaborative Research Board (CCRB) was formed and included representation from the study's target sample of women on probation and vertical stakeholders such as probation staff, service providers from the Community Court, substance use treatment providers, and IPV service providers.

Notably, the CCRB provided feedback on the design and implementation of Project WINGS to inform both arms of the intervention and improve the cultural appropriateness of the content and presentation.^29^

### Procedures

Research assistants recruited women from study sites by handing out flyers and inviting women to be screened. Eligible women who consented to participate completed screening and a baseline survey within 14 days of screening. Participant randomization occurred within 10 days of the baseline interview. Immediately after randomization, participants completed either the computerized WINGS or case manager WINGS. Finally, the study staff scheduled 3-month postintervention assessments with participants by text, e-mail, or phone.

All participants completed assessments using an audio computer-assisted self-interviewing (ACASI) system.^29^

### WINGS intervention

The computerized WINGS intervention was an applied IPV Screening, Brief Intervention, and Referral to Treatment (SBIRT) model,^30,31^ which provided a one-session, computerized self-paced assessment that allowed legal system-involved women who use drugs to (1) identify and disclose IPV, (2) provide feedback on their risks for IPV, (3) develop self-efficacy to protect themselves from IPV, (4) raise awareness of drug-related triggers for IPV, (5) develop safety plans considering substance-related risks for IPV, and (6) enhance social supports and linkages to IPV services. The social cognitive theory (SCT) guided the core components.^29,32^

The self-paced computerized tool was introduced to the participant by a case manager at the beginning of the program to help participants navigate through the tool. Computerized WINGS then guided participants through the tool with a culturally appropriate female narrator and a clickable audio button to read the text for each screen if preferred. The case manager version of WINGS provided the same core components delivered by a trained case manager.

Both WINGS conditions were conducted in a private room, and case managers were available to participants in both study arms to respond to questions after the session.^29^

### Measures

Participants were assessed at the baseline preintervention session and again at the 3-month follow-up session. In addition, researchers collected data on IPV and gender-based violence (GBV) in the past year.

#### Sociodemographics

Background variables included age (calculated as years from one's date of birth), race, education, employment status (unemployed yes/no), homeless in the past 90 days (yes/no), ever sentenced to prison (yes/no), and binge drinking (five or more drinks in 6 hours) in the past 90 days. Race was determined by responses from a survey question that asked respondents to check all races that applied to them: Black/African American, Hispanic/Latino(a), White/Caucasian, American Indian/Alaska Native, Asian/Southeast Asian/Pacific Islander, and/or Other.

For this analysis, we only included women who identified as Black/African American. We further stratified this group by women who only identified as Black/African American, those who identified as both Black/African American and Hispanic/Latina, and those who identified as Multiracial or Other, which included Black/African American and any other category (White/Caucasian, American Indian/Alaska Native, Asian/Southeast Asian/Pacific Islander, and/or Other).

Participants indicated their education level by choosing one of the following: no formal schooling, less than a high school diploma, high school diploma/GED, some college/two-year degree, four-year college degree, or postgraduate work. For this analysis, we created a dummy variable to indicate receipt of at least a high school diploma, where women with no formal schooling or less than a high school diploma were coded as 0 and all others were coded as 1 (having at least a high school diploma).

#### Social support

Social support was measured using six Likert-scaled items from the Enriched Social Support Inventory (ESSI) and a seventh non-Likert-scaled item (“Are you currently married or living with a partner?”).^33^ The non-Likert-scaled item was included in the analysis, but not in this variable since it is measured differently.

Our social support measure, including the Likert-scaled items, has a reliability of α = 0.86 and uses a 5-point Likert scale to assess the availability of emotional and instrumental support and advice for relationship conflicts or problems with intimate partners and other issues. The scale ranged from none of the time to all of the time. This measure was used to align with the parent study.^29^

#### IPV and GBV victimization

We used a shortened 15-item version of the revised Conflict Tactics Scale (CTS-2) and the Psychological Maltreatment of Women Inventory (PMWI) to assess IPV and GBV.^29,34,35^ Our instrument included eight items and four subscales from the CTS-2, dichotomized yes/no from the past year, measuring any (1) physical and injurious IPV (*i.e*., across severe and minor subscale items [combining]), (2) any sexual IPV (*i.e*., combining minor and severe subscale sexual IPV items), (3) any severe sexual IPV, and (4) a combined measure of severe verbal or psychological IPV (from the CTS-2 and PMWI).

Eight items from the PMWI were used to assess severe psychological abuse.^29^ Previous research indicated that internal consistency of the CTS-2 subscales ranges between α = 0.79 and α = 0.95,^34^ and for the PMWI scales, the value of α = 0.88.^35^

#### Drug use

Women reported drug use by responding to the following question: “In the past 30 days, how many days have you not used any drugs?”

#### Receipt of IPV services

Participants were asked one question: “Have you received any services, counseling, or group support for partner abuse in the past 90 days?”^29,31,36,37^

#### IPV prevention self-efficacy

Participants were assessed using the Domestic Violence Self-Efficacy Scale (DVSE), an 8-item scale with a reliability of α = 0.88.^38^ The DVSE assessed perceived competency in managing abuse and conflict with partners. Participants rated statements on a 5-point Likert scale, ranging from “never” to “always.”

### Statistical analyses

We calculated descriptive statistics using means/standard deviations (SDs) or frequencies/percentages of the sample's baseline demographic characteristics and outcome measures by study arm. We employed an intent-to-treat approach and used multilevel mixed-effects models to test intervention effects. Multiple imputation was used to impute values for missing data by using the information we observed or measured at prior assessments to predict values for missing variables.^39^

**FIG. 1.**
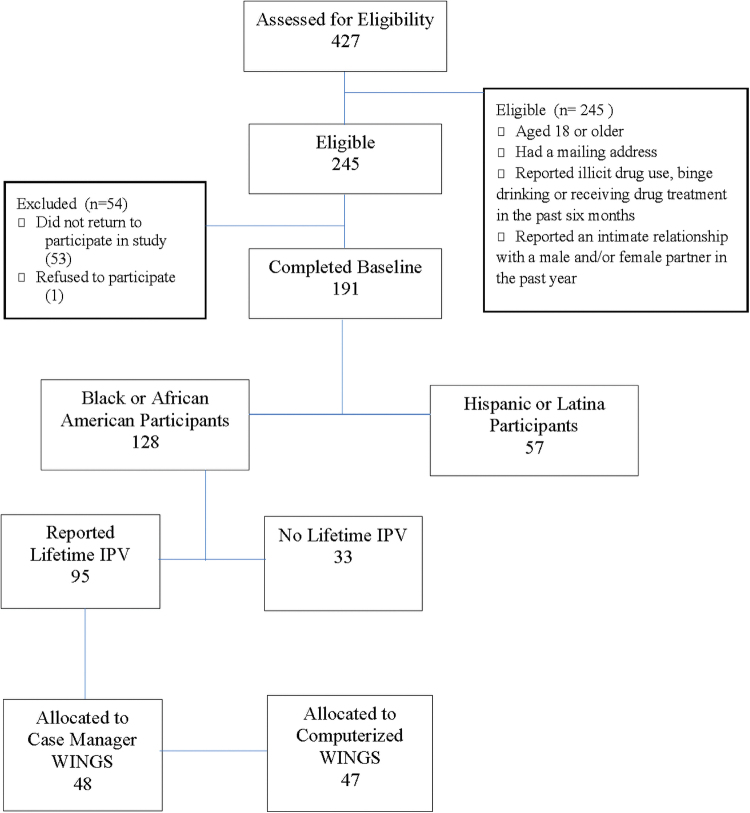
WINGS participant allocation for subgroup analysis. IPV, intimate partner violence; WINGS, Women Initiating New Goals of Safety. This figure uses content published by Gilbert et al.^29^

The imputation procedures were performed on data from the parent study and then we selected a sample for subgroup analysis in this study.^29^ In the parent study, the missingness was due to loss to follow-up, and the missing rate was 10%. The subgroup sample comprised only Black WINGS participants (*n* = 128) and was further stratified to focus on women with a history of IPV at baseline (*n* = 95) (Fig. 1).

We calculated descriptive statistics using percentages, SDs, and frequencies of the sample's baseline demographic characteristics and outcome measures by study arm. To estimate the intervention effects, multilevel mixed-effects models were employed. The models included treatment conditions, follow-up time and the interaction terms between conditions and follow-up time, participant's reports of a history of IPV, and covariate adjustments for baseline measures. The models also set repeated measures and study sites as random effects.

Intervention effects are reported as follows: adjusted odds ratio (aOR) using mixed-effects logistic regression for received IPV services (past 90 days); regression coefficient (*b*) using mixed-effects linear regression for IPV self-efficacy/social support; and incident rate ratios (IRRs) using mixed-effects Poisson regression for days not using drugs (past 30 days).

Statistical significance was assessed at the *p* < 0.05 level. All analyses were conducted in Stata 15.

### Human participant protection

The Institutional Review Boards (IRBs) at Columbia University and the Center for Court Innovation approved the study protocols before implementation, and researchers adhered to these study protocols. In addition, we obtained and archived written informed consent from participants. Trial registration can be found on clinicaltrials.gov using the following identifier: NCT01788579.

## Results

### Parent study participants

Two probation sites and a community court-administered alternative-to-incarceration program were chosen as participant recruitment sites. Figure 1 shows that between May 2012 and January 2013, 427 women were recruited and screened for the following inclusion criteria: (1) aged 18 years or older; (2) reporting illicit drug use, binge drinking, or receiving drug treatment in the past 6 months; (3) reporting an intimate relationship with a male and/or female partner in the past year; and (4) having a mailing address.

Participants received $100 for completing the baseline and 3-month follow-up assessments and attending the intervention session. Of those initially recruited, 245 women met eligibility criteria, and 191 women who consented to participate enrolled in the project. The retention rate was 89.5%, with 171 women completing the 3-month follow-up assessment. All women were randomized to receive either the computerized (*n* = 94) or case manager-delivered (*n* = 97) WINGS intervention.^29^

As reported in the parent study, there were no differences in outcomes between study arms (computerized vs. case manager groups), suggesting that both were effective.^29^ Specifically, there were no significant differences in sociodemographic characteristics or primary baseline measures (*i.e*., CTS-2 and single-item assessing receipt of IPV services) or secondary outcomes (*i.e*., IPV prevention self-efficacy, social support, and days of abstinence from drug use) among participants in both groups.

In both arms, participants received an initial psychosocial education session on rates of IPV in the United States, a review of the types of IPV, and a description of drug-related triggers for IPV.^29^

### Participant characteristics in the subanalysis

Data were analyzed for the subgroup of 128 Black female participants, 95 of whom reported lifetime IPV. Table 1 presents demographic and baseline characteristics across both study arms. Participants were on average 33 years of age (SD: 11.5). While all women identified as Black or African American, some women also identified as Hispanic or Latina (8.6%) or as multiracial or other races (9.6%). Most women were single (74.2%) and unemployed (82.8%).

**Table 1.** Baseline Characteristics of Study Sample (*N* = 128)

	Black/African American women (*n* = 128)
Age, mean (SD)	33.4 (11.5)
Black or African American, *n* (%)	128 (100)
Hispanic or Latina, *n* (%)	11 (8.6)
Multiracial or Other, *n* (%)	12 (9.4)
Less than high school diploma, *n* (%)	46 (35.9)
Single, never married, *n* (%)	96 (74.2)
Homelessness, past 90 days, *n* (%)	14 (20.3)
Unemployment (current), *n* (%)	106 (82.8)
Sentenced to prison, *n* (%)	22 (16.4)
Substance use behaviors, *n* (%)	
Crack/cocaine use, ever	34 (26.5)
Binge drinking, ever	79 (61.7)
Injection drug use, past 90 days	12 (9.3)
Experiences of violence (as measured by CTS-2), *n* (%)	
Any physical IPV	55 (42.9)
Any sexual IPV	39 (30.40)
Any physical or sexual IPV	63 (49.2)
Severe verbal or psychological IPV (as measured by CTS-2 and PMWI)	93 (72.6)

CTS-2, Revised Conflict Tactics Scale; IPV, intimate partner violence; PMWI, Psychological Maltreatment of Women Inventory; SD, standard deviation.

A third of the participants (35.9%) had less than a high school diploma, about a fifth (20.3%) were homeless in the past 90 days, and some (16.4%) were sentenced to prison at some point in their lives. The percentage of participants reporting binge drinking ever was high (61.7%). Some participants (26%) in our sample reported ever using crack/cocaine. Only 9.3% reported injecting drugs in the past 90 days.

Experiences of violence were common, with 42.9% reporting any physical or sexual IPV and 72.6% reporting severe verbal or psychological IPV in the 12 months before the survey.

### Effects of the intervention

At the 3-month follow-up assessment (Table 2), women in the case manager arm who experienced physical, sexual, and/or psychological IPV were 14 times more likely to receive IPV services in the past 90 days—from baseline to the 3-month follow-up (aOR = 14.45, 95% confidence interval [CI] = 1.25 to 166.51, *p* = 0.032). Black or African American women in the computerized arm reported a significant increase in social support at the 3-month follow-up compared with baseline (*b =* 2.27, 95% CI = 0.43 to 4.11, *p* = 0.015).

**Table 2.** Effects of Computerized and Case Manager Women Initiating New Goals of Safety Interventions on Linkage to Intimate Partner Violence Services and Secondary Outcomes Among Black Participants Who Experienced Physical, Sexual, and Psychological Intimate Partner Violence

	Arm	Total sample, *n* (%) or mean (SD)		Results from multilevel mixed-effects model with random effects set for repeated measures and sites		
		Baseline	3 Months	Effect estimate	[95% CI], *p* value	Difference of change between case manager and computerized arms
		*N* = 95	*N* = 85			
Received IPV services (past 90 days): OR	Case manager	1 (2.1%)	7 (16.3%)	**14.45** ^a^	**[1.25 to 166.51], 0.032**	0.18 [0.01 to 2.84], *p* = 0.222
	Computerized	5 (10.6%)	8 (19.1%)	2.59	[0.59 to 11.13], 0.206	
IPV self-efficacy: *b*	Case manager	21.88 (5.84)	23.37 (5.86)	1.40	[−0.80 to 3.61], 0.212	0.70 [−2.45 to 3.84], *p* = 0.664
	Computerized	20.89 (6.96)	22.81 (6.07)	2.10	[−0.16 to 4.36], 0.069	
Social support: *b*	Case manager	20.65 (5.98)	21.93 (5.33)	1.36	[−0.39 to 3.10], 0.127	0.91 [−1.63 to 3.45], *p* = 0.480
	Computerized	20.11 (6.78)	22.26 (6.39)	**2.27** ^a^	**[0.43 to 4.11], 0.015**	
Days not using drugs (past 30 days): IRR	Case manager	13.00 (12.98)	17.98 (12.89)	**1.37^b^**	**[1.20 to 1.58], <0.001**	0.88 [0.73 to 1.06], *p* = 0.174
	Computerized	14.65 (13.29)	17.98 (12.72)	**1.21** ^a^	**[1.04 to 1.39], 0.012**	

Values in bold indicate statistical significance at the 0.05 or 0.01 level.

^a^ *p* < 0.05 and ^b^*p* < 0.01.

*b*, Regression coefficient; CI, confidence interval; IRR, incident rate ratio; OR, odds ratio.

Participants in both arms had significantly more days of abstinence from drug use at the 3-month follow-up compared with the baseline assessment (IRR = 1.37, 95% CI = 1.20 to 1.58, *p* < 0.001, in the case manager arm; and IRR = 1.21, 95% CI = 1.04 to 1.39, *p* = 0.012, in the computerized arm). Neither study arm showed a significant change in IPV self-efficacy from baseline to follow-up. In addition, the difference in change between the two study arms was not significant for any outcome.

## Discussion

In the parent study (including all races), both WINGS conditions saw significant improvements in all outcomes over the 3-month follow-up period, with no differences across study arms.^29^ Our subanalysis of only Black women who experienced IPV at baseline found significantly increased number of days of abstinence from drug use in both formats.

We had mixed results regarding the receipt of IPV services and social support. Only the case manager arm experienced statistically significant improvements in IPV services over the 3-month follow-up period, and only the computerized group had improvements in social support. We found no statistically significant improvements in IPV self-efficacy across either study arm.

However, for all outcomes, there were observable improvements across both arms from baseline to the 3-month follow-up, but no significant differences were found between the study arms. The sample size may not have been large enough to detect a small effect size.

Similar to the parent study, findings showed that both modalities of WINGS had promise in identifying and addressing IPV victimization among Black women in community supervision programs who use drugs or engage in binge drinking. In this subanalysis, the case manager approach worked well for increasing receipt of IPV services, whereas the computerized approach was conducive to increasing social support.

Several factors may explain the differential effectiveness of the computerized versus case manager modalities in improving social support and linkage to services, including differential fidelity in delivering social support and linkage to services (*e.g*., the social support mapping exercise entails multistep visual interactive mapping that may have had better fidelity in implementation in the computerized modality vs. the case manager modality) and the relative importance of having in-person engagement, coaching, and support with the case manager modality.

Therefore, this may have impacted whether participants sought out and received social support if assigned to the computerized arm. Additionally, having in-person engagement, coaching, and support from the case manager modality may have contributed to the receipt of IPV services for participants in the case manager modality. In addition, the lack of significant improvement in social support found for the case manager group and lack of improvement in linkage to services for the computerized group may be due to the relatively small sample size and lack of sufficient power. These findings suggest that different WINGS modalities may work better for different activities and point to the need for hybrid formats that optimize the use of different modalities.

Furthermore, future culturally tailored adaptations of WINGS might benefit from recognizing the strengths of social support among Black women and selecting culturally tailored strategies for linking Black women to competent service providers. Such culturally tailored adaptations should be guided by Black women with lived experience of the criminal legal system and IPV and robust community engagement of key stakeholders in the Black community who may support Black women in the development stage.

Racial health equity frameworks should guide implementation research for delivering culturally tailored versions of WINGS.^40^

### Limitations

Due to the small sample size and how the data were collected, our findings are not generalizable to the entire population of Black women who use drugs or engage in binge drinking and are involved in community supervision settings. The study did not use an attentional control group; therefore, it was difficult to know if treatment gains for both conditions were because of the WINGS intervention or outside factors.

Additionally, reporting of IPV victimization during the intervention was measured for two different time frames—the past year and the past 3 months. These factors precluded the assessment of IPV as a study outcome. Finally, although study findings show that WINGS effectively identified and addressed IPV among this subgroup of Black women, the intervention was not specifically tailored for Black women.

Future intervention research guided by racial health equity frameworks with more robust community engagement may further tailor and optimize both the modalities and content of the WINGS intervention core components for Black women. It is important to note that some data show that female-to-male IPV occurs more frequently than male-to-female IPV. Our study did not include male participants.

Despite this study's limitations, its noteworthy strengths were a high retention rate (89.5% at the 3-month follow-up), high fidelity of implementation in both conditions, and randomization of the two interventions with blind assessment of outcomes.^41^

## Conclusions

Consistent with previous research, the high rates of IPV found among this sample of Black women in community supervision programs who use drugs underscore the urgent need for scaling up integrated IPV prevention interventions. Survivors of IPV, particularly those with co-occurring SUDs and who are also involved in the criminal legal system, could benefit from a culturally sensitive SBIRT intervention such as WINGS.

We further suggest focusing resources to meet the needs of Black women survivors to amplify their access to social support and social networks. Outreach should specifically consider culturally appropriate ways to engage Black women at risk of legal involvement and link them to strategies that manage and reduce exposure to IPV risks and enhance social support networks.

The recent spike in rates of IPV and substance use during the COVID-19 pandemic, coupled with the pre-existing overrepresentation of Black women in the criminal legal system due to racialized drug laws and policing practices,^42^ underscores the need to use evidence-based practices that effectively address Black women's exposure to violence.^43^

Additionally, the pandemic has made escaping IPV more challenging due to increased vulnerability to social isolation; increased anxiety related to health concerns; exacerbated mental health crises; reduced access to mental health services; increased financial, food, and housing insecurity; and reduced access to first responders.^44–46^ These factors increase the incidence of IPV and tend to increase the severity of IPV among women at greater risk.^45^

Despite these concerns, no IPV SBIRT interventions are tailored to Black women with multiple intersecting identities. Future adaptations should consider this tool's cultural appropriateness and intersectionality, potentially enhancing results if it is developed by Black women impacted by the criminal legal system.^1,47^ Such an embedded development process may increase its acceptance and effectiveness among Black and Brown women in the criminal legal system.^48^

Importantly, if IPV services are expanded without focusing on strategies to improve social support, we may see an increase in IPV prevention, but achieve relatively little impact on the IPV outcomes of individuals currently experiencing IPV. Opportunity for telehealth adaptation of IPV tools and interventions for Black women may provide promising results. Offering either version of WINGS in this way may facilitate provision of IPV prevention and social support tools to more women in the most vulnerable communities.^49^

The shortage of culturally sensitive interventions and the lack of trusted relationships with police and service providers also pose challenges in accessing services. Furthermore, traditional forms of IPV reporting in community settings, such as reporting to a case manager, versus more confidential forms of reporting, such as WINGS, may need further modification to improve linkage to care. Targeted investments in IPV services and specific culturally tailored interventions for Black women may improve the rates of IPV service utilization and cost-effectiveness of care.

WINGS has been culturally adapted in several world regions using local partnerships. This adaptation is imperative to building trust, especially among Black people with a long history of mistrust, when seeking and receiving police services and medical care. By expanding access to interventions with demonstrated value, such as WINGS, we enhance outreach to those most in need of time-sensitive public health interventions to engage society's most vulnerable.

## Data Access Statement

Data for this study will be made available after approval of the proposal by the Principal Investigators or a designated representative.

## Abbreviations Used

aOR: adjusted odds ratio
*b*: regression coefficient
CCRB: Community Collaborative Research Board
CI: confidence interval
CTS-2: Revised Conflict Tactics Scale
DVSE: Domestic Violence Self-Efficacy Scale
GBV: gender-based violence
IPV: intimate partner violence
IRR: incident rate ratio
NIDA: National Institute on Drug Abuse
OR: odds ratio
PMWI: Psychological Maltreatment of Women Inventory
SBIRT: Screening, Brief Intervention, and Referral to Treatment
SD: standard deviation
SUD: substance use disorder
WINGS: Women Initiating New Goals of Safety
